# Inanspruchnahme und Ausgaben in der zahnmedizinischen Versorgung: Eine Übersicht auf Basis von Routinedaten aus Deutschland

**DOI:** 10.1007/s00103-021-03374-1

**Published:** 2021-07-05

**Authors:** Michael H. Walter, Michael Rädel

**Affiliations:** Poliklinik für Zahnärztliche Prothetik, Medizinische Fakultät Carl Gustav Carus, Technische Universität Dresden, Fetscherstraße 74, 01307 Dresden, Deutschland

**Keywords:** Zahnärztliche Versorgung, Inanspruchnahme, Ausgaben, Therapie, Prävention, Dental care, Utilization, Expenses, Treatment, Prevention

## Abstract

**Hintergrund:**

Inanspruchnahme und Ausgaben gehören zu den wichtigsten Kenngrößen von Versorgungssystemen. Sie erlauben auch Rückschlüsse auf die Ausrichtung der praktizierten Zahnmedizin.

**Ziel der Arbeit:**

Ziel dieser Übersicht ist es, den Status quo der Inanspruchnahme zahnärztlicher Versorgung in Deutschland und der diesbezüglichen Ausgaben anhand vorhandener Routinedaten aus der vertragszahnärztlichen Versorgung zu beschreiben.

**Material und Methoden:**

Die zugrunde liegenden Analysen wurden den jährlichen Zahnreporten der gesetzlichen Krankenkasse BARMER entnommen. Sie basieren auf deutschlandweiten Abrechnungsdaten von 2010 bis 2018.

**Ergebnisse:**

Die Inanspruchnahme zahnärztlicher Leistungen liegt in Deutschland mit jährlichen Raten um 70 % überdurchschnittlich hoch. Für Füllungen lag die Inanspruchnahmerate 2018 in den Altersgruppen zwischen 30 und 79 Jahren durchgehend um 30 %, für Neueingliederungen von Zahnersatz und Zahnkronen in den Altersgruppen von 55 bis 84 Jahren bei mindestens 10 %. Im Bereich der Prävention war ein kontinuierlicher Anstieg für Früherkennungsuntersuchungen bei Kindern zu verzeichnen (31,9 % in 2010, 35,2 % in 2018). Bei individualprophylaktischen Maßnahmen bei 6‑ bis 17-Jährigen war kein klarer Trend erkennbar (64,0 % in 2010, 65,4 % in 2018). Die Ausgaben der gesetzlichen Krankenkasse stiegen moderat.

**Diskussion:**

Durch die hohe Inanspruchnahme sind gute Voraussetzungen für eine präventionsorientierte Zahnmedizin gegeben. Wenn auch eine positive Tendenz zu einer leicht steigenden Inanspruchnahme präventiver Leistungen erkennbar ist, bildet sich im Versorgungsgeschehen nach wie vor eine mehrheitlich auf invasive Intervention orientierte Zahnmedizin ab. Als sinnvolle Instrumente zur Umsetzung eines substanziellen Paradigmenwechsels können unter anderem eine weitere Förderung der Gesundheitskompetenz der Bevölkerung und präventionsfördernde Anreize angesehen werden.

## Abstract (en)

**Background:**

Utilization and expenses are among the most important parameters of healthcare systems. Moreover, they allow us to draw conclusions about the features and focus of currently practiced dentistry in Germany.

**Objectives:**

The aim was to describe the current status of the utilization of dental services and the related expenses in Germany based on routine data from the statutory health insurance system.

**Materials and Methods:**

The underlying analyses were taken from the annual dental reports of the statutory health insurance company BARMER. They were based on Germany-wide claims data from 2010 to 2018.

**Results:**

The utilization of dental services in Germany with annual rates around 70% is distinctly above average. In 2018, utilization rates for dental fillings in the age groups between 30 and 79 years were consistently around 30% and for new prosthetic restorations and dental crowns in the age groups between 55 and 84 years it was at least 10%. In the field of prevention, there has been a steady increase of screening exams for children (31.9% in 2010 and 35.2% in 2018). For individual prophylactic measures in 6‑ to 17-year-olds, no clear trend was discernible (64.0% in 2010 and 65.4% in 2018). The expenses of the statutory health insurance increased moderately.

**Conclusions:**

With the high utilization rate, good prerequisites for prevention-oriented dentistry exist. Although there is a positive trend towards a slight increase in the utilization of preventive services, dental care is still mostly oriented towards invasive intervention. Further promotion of health literacy of the population and incentives promoting prevention can be seen as useful instruments to implement a substantial paradigm shift.

## Einleitung

Inanspruchnahme und Ausgaben gehören zu den wichtigsten Kenngrößen von Versorgungssystemen. Sie zeigen Auswirkungen struktureller Merkmale des Gesundheitssystems auf und erlauben Rückschlüsse auf den Zugang zur Versorgung, soziale Ungleichheit, Morbidität und das Gesundheitsbewusstsein der Bevölkerung. Sie ermöglichen auch vertiefende Analysen zur Verteilung der eingesetzten Ressourcen. Die Auswertung von Routinedaten von Krankenkassen ist eine von verschiedenen Möglichkeiten der Erfassung. Die Ergebnisse der Analysen können für Entscheidungen zur Ausrichtung des Systems, Lenkung der Ressourcen und Bedarfsplanung genutzt werden.

Im Jahr 2019 stellte eine Veröffentlichung im renommierten Journal *The Lancet* der heutigen zahnmedizinischen Versorgung aus einer globalen Perspektive ein schlechtes Zeugnis aus [1]. Die Autoren forderten darin unter anderem die radikale Abkehr von einem zunehmend auf Intervention, Hightech und Ästhetik fokussierten Ansatz, der durch Profitdenken und Konsumismus gefördert werde. Auch wenn sich die Zahnmedizin international dieser Problematik bewusst sei, würden weiterhin vermehrt Interventionen „downstream“, also erst nach bereits erfolgter Erkrankung erfolgen. Die Zahnmedizin müsse sich breiter präventionsorientiert aufstellen, public-health-basierter werden und multisektoral zusammenarbeiten, um den globalen Herausforderungen oraler Erkrankungen erfolgreich entgegentreten zu können.

Wo stehen wir in Deutschland? Die vertragszahnärztliche Versorgung in Deutschland verfügt über eine hohe Akzeptanz in der Bevölkerung. Sie bietet den Versicherten im internationalen Vergleich ein sehr breites Leistungsspektrum auf hohem Niveau an. Leistungen, wie z. B. hochwertiger festsitzender Zahnersatz, sind in vielen westlichen Industrieländern keine gesetzlichen Gesundheitsleistungen. Auf dieser Grundlage riefen die kritischen Töne der *Lancet*-Publikation vielfache Reaktionen hervor [2]. Dabei stand die Frage im Mittelpunkt, ob sich die deutsche Zahnmedizin nicht schon längst in diesem Paradigmenwechsel von der therapie- hin zur präventionsorientierten Zahnmedizin befindet oder die Kritik berechtigt ist.

Ziel dieser Publikation ist nicht nur die exemplarische Darstellung von ausgewählten Inanspruchnahmen und Ausgaben in der deutschen vertragszahnärztlichen Versorgung auf Basis von Routinedaten, sondern auch der Versuch einer Standortbestimmung in Bezug auf den geforderten Paradigmenwechsel. Das diesbezügliche Potenzial von Routinedatenanalysen soll an Beispielen aufgezeigt werden.

## Methodik der Sekundärdatenanalyse

Basis für die nachfolgende Analyse von Inanspruchnahme und Ausgaben in der zahnärztlichen Versorgung in Deutschland waren die Ergebnisse der BARMER-Zahnreporte aus den Jahren 2014 bis 2020 [3–9]. Die hier zugrunde liegenden Daten beziehen sich überwiegend auf den Zeitraum von 2010 bis 2018. Lediglich die Inanspruchnahmeraten von Parodontitisdiagnostik/-therapie sowie die Inanspruchnahmeraten von Präventionsmaßnahmen bei Pflegebedürftigen beziehen sich auf die Jahre 2012 bis 2018 bzw. 2012 bis 2016. Diese Ergebnisse wurden jährlich alters- und geschlechtsadjustiert auf die deutsche Gesamtbevölkerung ausgewiesen und beruhten auf den Abrechnungsdaten von jeweils ca. 9 Mio. Versicherten der BARMER, also etwa 11 % der deutschen Bevölkerung. Datengrundlage waren im Wesentlichen die gemäß den Gebührenpositionen des einheitlichen Bewertungsmaßstabes für zahnärztliche Leistungen (BEMA) abgerechneten Leistungen und im Bereich „Zahnersatz und Zahnkronen“ die abgerechneten Festzuschüsse. Für den BEMA-Teil 1 waren maschinell auswertbare Daten seit 2010 verfügbar, für die BEMA-Teile 2 bis 5 seit 2012. Für weitere Details wird auf die BARMER-Zahnreporte verwiesen [3–9].

## Gesamtinanspruchnahme

Die Gesamtinanspruchnahme zahnärztlicher Versorgung lässt sich mit nur geringer Unschärfe über die Inanspruchnahme von Leistungen des Teils 1 des BEMA abbilden, der diagnostische und Beratungsleistungen umfasst, die fast in jedem Behandlungsfall erforderlich sind. Die versichertenbezogenen Inanspruchnahmeraten blieben in Deutschland über die letzten Jahre hinweg stabil in einem Bereich von 70 % (Tab. 1).

**Table 1:** 

	Versichertenbezogene jährliche Inanspruchnahmerate in Prozent								
	2010	2011	2012	2013	2014	2015	2016	2017	2018
Gesamt (BEMA Teil 1)^a^	70,5	70,8	69,9	70,9	71,3	71,0	70,9	70,9	70,2
Füllungen^a^	29,6	29,2	28,2	29,1	28,8	28,4	27,8	27,3	26,5
Röntgenpanoramaschichtaufnahmen^a^	8,7	8,8	8,6	8,9	9,2	9,2	9,3	9,4	9,6
Wurzelkanalbehandlungen^a^	6,4	6,3	6,1	6,0	6,0	5,8	5,6	5,5	5,3
Extraktionen^a^	9,5	9,4	9,1	9,0	9,0	8,8	8,7	8,5	8,3
Parodontitisdiagnostik (PSI) und -therapie^a^	–	–	24,6	24,3	24,6	25,3	25,8	26,0	26,9
Früherkennungsuntersuchung (Kinder < 6 Jahre)^a^	31,9	32,1	32,4	33,5	33,9	34,7	35,1	35,9	35,2
Individualprophylaxe (Kinder und Jugendliche 6 bis < 18 Jahre)^a^	64,0	64,6	64,0	64,0	64,5	65,9	65,7	65,9	65,4
Diagnostische und präventive Maßnahmen (vollstationär Pflegebedürftige ab 65 Jahre)^b^	–	–	39,2	39,2	39,0	38,0	38,1	–	–
Diagnostische und präventive Maßnahmen (ambulant Pflegebedürftige ab 65 Jahre)^b^	–	–	54,5	55,0	55,6	55,7	55,8	–	–

^a^einheitlich auf die Alters- und Geschlechtsstruktur der Bevölkerung Deutschlands zum 31.12.2011 standardisiert

^b^einheitlich auf die Alters- und Geschlechtsstruktur der Bevölkerung Deutschlands zum 31.12.2012 standardisiert

Eine internationale Einordnung dieser Inanspruchnahmeraten erscheint vor dem Hintergrund unterschiedlichster Gesundheits- und Versorgungssysteme und einer sehr eingeschränkten Verfügbarkeit vergleichbarer Daten schwierig. Selbst innerhalb Europas sind die Unterschiede erheblich. Für die Niederlande wurde eine etwas höhere jährliche Inanspruchnahmerate von 80 % für 2018 beschrieben, basierend auf einem positiven Trend beginnend mit 74 % im Jahr 1996 [10]. Im Gegensatz dazu lassen ältere Ergebnisse aus Finnland auf eine jährliche Inanspruchnahme von nur etwa 38 % schließen [11]. Studienergebnisse aus Bosnien-Herzegowina beschrieben eine jährliche Inanspruchnahmerate von nur 20 % [12]. Im weltweiten Vergleich liegen die deutschen Inanspruchnahmeraten in der Spitzengruppe. Eine aktuelle Untersuchung aus Japan wies die jährliche Inanspruchnahme mit Werten zwischen 38 % und 42 % für kurative Behandlungen und zwischen 23 % und 33 % für präventive Maßnahmen aus [13]. Für die USA beschrieb die American Dental Association die Inanspruchnahmeraten im Jahr 2014 mit 48 % bei Kindern, 36 % bei Erwachsenen im berufstätigen Alter und 44 % bei den Senioren [14]. Insgesamt erscheint es daher relativ sicher, die Gesamtinanspruchnahme zahnärztlicher Versorgung in Deutschland im internationalen Kontext als vergleichsweise hoch zu bewerten.

**Figure 1:**
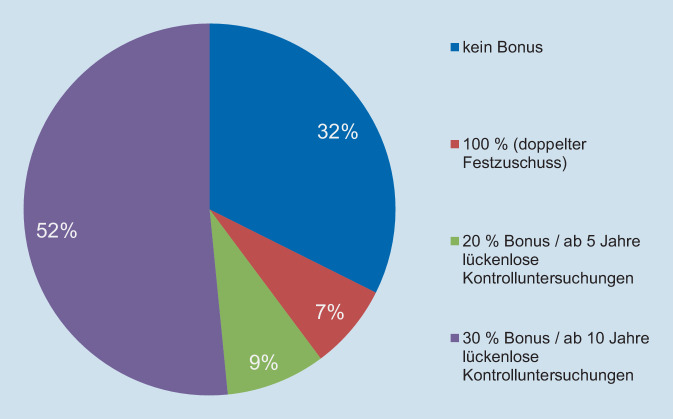

Ein wesentlicher Grund für die hohen Inanspruchnahmen dürfte in einem relativ gut entwickelten Gesundheitsbewusstsein liegen. In der Fünften Deutschen Mundgesundheitsstudie (DMS V) wurde eine gute Ausprägung der Selbstwirksamkeitsüberzeugung zur eigenen Zahngesundheit festgestellt [15]. Drei Viertel der DMS-Studienteilnehmenden gingen davon aus, selbst „viel“ oder „sehr viel“ zum Erhalt der eigenen Zähne beitragen zu können. In der Altersgruppe der jüngeren Senioren konnte diesbezüglich sogar ein Anstieg von 67 % im Jahr 2005 auf 76 % im Jahr 2014 ermittelt werden [15]. Darin und in einem verbesserten Mundgesundheitsverhalten (Zähneputzen, kontrollorientierte Zahnarztbesuche) ließ sich ein wachsendes Mundgesundheitsbewusstsein in dieser Altersgruppe erkennen. Weitere Ursachen sind in dem einfachen Zugang zur Versorgung, der Kostenfreiheit von Vorsorgeuntersuchungen und vermutlich dem Bonussystem für Zahnersatz zu sehen, das allerdings eher ab einer mittleren Altersgruppe Bedeutung erlangen dürfte. Etwa 2 Drittel der Inanspruchnehmenden von Zahnkronen- und Zahnersatzleistungen nutzten die Bonusoption und haben in diesem Zusammenhang Anspruch auf eine Zuschusserhöhung, d. h., sie konnten über mindestens 5 Jahre jährliche Vorsorgeuntersuchungen nachweisen (Abb. 1). Für das Bonussystem spricht vor allem der Anreiz zu einer kontrollorientierten Inanspruchnahme.

Im internationalen Vergleich spielt die Kostenfreiheit von Vorsorgeuntersuchungen eine wichtige Rolle. Die vergleichsweise hohe Zahnarztdichte in Deutschland von einem behandelnd tätigen Zahnarzt auf 1146 Einwohner ermöglicht zudem einen einfachen Zugang zu zahnärztlicher Versorgung [16]. Im Vergleich dazu lag 2007 das Verhältnis aktiver Zahnarzt zu Einwohner in den Staaten der Europäischen Union (EU) und der Schweiz bei 1:1500 [17]. Weltweit war der Zugang zur zahnärztlichen Versorgung zum Teil deutlich schwieriger und/oder deutlich einkommensabhängiger [18].

## Inanspruchnahme Füllungen

**Figure 2:**
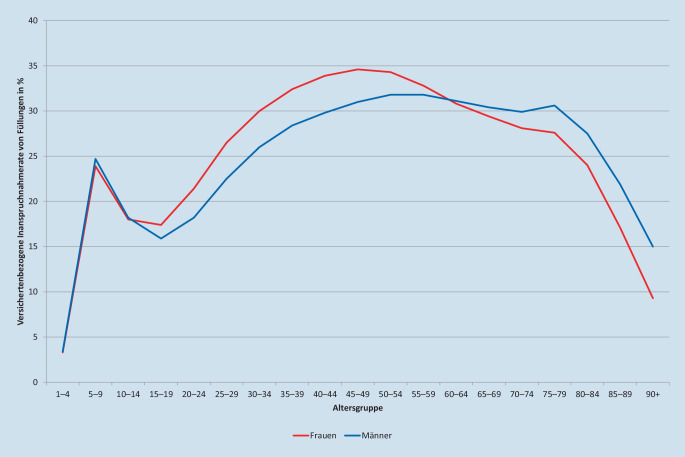

Bei den Zahnarztkontakten zeigte sich eine relativ hohe Inanspruchnahme von invasiven Therapieleistungen, die in der Praxis nach wie vor einen großen Stellenwert einnehmen. Als Indikator für die Häufigkeit invasiver Interventionen eignete sich die Füllungstherapie, besonders in den jüngeren Altersgruppen. Die tatsächliche Inanspruchnahme invasiver Leistungen ist höher, da unter anderem parodontaltherapeutische, chirurgische und prothetische Leistungen ebenfalls einbezogen werden müssten. Für Füllungsleistungen lag 2018 die jährliche versichertenbezogene Inanspruchnahmerate in den Erwachsenenaltersgruppen zwischen 30 und 79 Jahren durchgehend um 30 % mit einem Gipfel von 35 % bei 45- bis 49-jährigen Frauen (Abb. 2). Über alle Altersgruppen betrug der Anteil der Inanspruchnehmenden von Füllungen an den Inanspruchnehmenden von zahnärztlichen Leistungen insgesamt etwa 38 %. Im Jahr 2010 lag dieser Wert noch bei etwa 42 %. Dies kann als Hinweis auf einen positiven Trend gewertet werden. Aus der Sicht der Autoren sind diese Werte aber unerwartet hoch. Der Zustand der versorgten Zähne war uns aus den Abrechnungsdaten nicht bekannt. Es handelte sich um sogenannte abgerechnete Morbidität [19, 20]. Die Ergebnisse unterlagen damit den bekannten Unsicherheiten, beispielsweise durch mögliche Dokumentations- und Abrechnungsfehler [21]. Trotzdem liegt die Vermutung nahe, dass bei einem erheblichen Anteil der Versicherten längere therapiefreie Phasen nicht erreicht wurden. Allerdings ist auch festzustellen, dass bei der jährlichen Inanspruchnahme von Füllungen zwischen 2010 (29,6 %) und 2018 (26,5 %) ein positiver, leicht rückläufiger Trend erkennbar wurde (Tab. 1). Dieses Ergebnis steht in Übereinstimmung mit Daten der Kassenzahnärztlichen Bundesvereinigung, die zwischen 1991 und 2019 einen weitgehend kontinuierlichen Rückgang der abgerechneten Füllungen zeigten [16]. Dabei ist allerdings zu berücksichtigen, dass hier kein direkter Vergleich zu den Inanspruchnahmeraten pro versicherte Person möglich ist.

## Inanspruchnahme weiterer Schlüsselmaßnahmen

Die Röntgenpanoramaschichtaufnahme kann als ein Indikator für diagnostischen Aufwand gewertet werden. Zwischen 2010 (8,7 %) und 2018 (9,6 %) war eine kontinuierliche Zunahme der jährlichen Inanspruchnahmeraten zu verzeichnen (Tab. 1; [4]). Eine Einordnung dieser Werte fällt schwer, da keine internationalen Vergleichszahlen verfügbar sind. Zu den Gründen der Zunahme können keine tragfähigen Hypothesen formuliert werden.

Wurzelkanalbehandlungen zeigen häufig einen schwerwiegenden Verlauf einer Karieserkrankung an. Zwischen 2010 (6,4 %) und 2018 (5,3 %) war ein kontinuierlicher Rückgang der jährlichen Inanspruchnahmeraten feststellbar [4]. Dieser Trend könnte durch eine abnehmende Karieslast und frühere, sekundärpräventiv wirksame Intervention zustande kommen. Zahnextraktionen werden in den meisten Fällen im Finalstadium von Zahn- und Zahnbetterkrankungen durchgeführt. Zwischen 2010 (9,5 %) und 2018 (8,3 %) war ein kontinuierlicher Rückgang der jährlichen Inanspruchnahmeraten zu verzeichnen [4]. Auch dieser Trend deutet auf eine sich verbessernde Mundgesundheit hin. Allerdings ist auch hier aufgrund möglicher Störgrößen eine vorsichtige Interpretation angezeigt. Mögliche Störgrößen sind beispielsweise sich ändernde Präferenzen der Behandelten und ein verändertes Extraktionsverhalten der Behandelnden. Der rückläufige Trend bei Wurzelkanalbehandlungen und Extraktionen bildet sich auch in den Daten der Kassenzahnärztlichen Bundesvereinigung ab [16], allerdings bei anderem Auswertungsansatz (s. oben).

Die gemeinsame jährliche Inanspruchnahmerate parodontaldiagnostischer und -therapeutischer Leistungen zeigte zwischen 2012 (24,6 %) und 2018 (26,9 %) einen Anstieg. Den weit überwiegenden Anteil machte dabei der parodontale Screeningindex (PSI, BEMA Teil 1, Nr. 04) als parodontaldiagnostische Leistung aus. Wird berücksichtigt, dass der PSI durch gesetzlich Versicherte alle 2 Jahre in Anspruch genommen werden kann, ergibt sich eine Schätzung von knapp 50 % der theoretisch möglichen Ausschöpfung. Dagegen war die jährliche Inanspruchnahmerate bei parodontaltherapeutischen Leistungen mit 1,8 % (2018) vor dem Hintergrund der aus epidemiologischen Studien bekannten hohen Prävalenzen niedrig. In der DMS V beispielsweise lag die Prävalenz schwerer Parodontalerkrankungen bei 65- bis 74-Jährigen je nach Klassifikationssystem bei 24,6 % bzw. 19,8 % [15].

**Figure 3:**
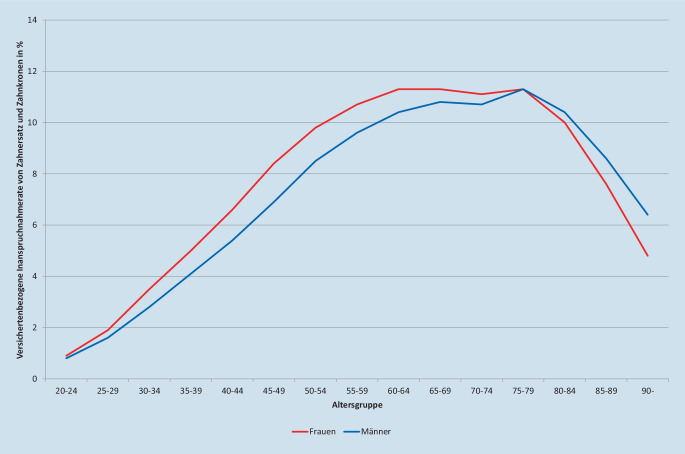

Die Inanspruchnahmerate von neuem Zahnersatz und neuen Zahnkronen lag 2018 bei 7,4 %, mit Werten von mindestens 10 % in den Altersgruppen von 55 bis 84 Jahren (Abb. 3; [4]). Die Verteilung der Inanspruchnahmeraten über die Altersgruppen widerspiegeln die aus Bedarfsanalysen bekannten kontinuierlichen Anstiege mit dem Alter [22], hier allerdings mit klarem Trend nur bis etwa 60 Jahre, um ab 80 Jahren wieder deutlich zu fallen.

## Inanspruchnahme von Präventionsleistungen (Prophylaxeleistungen)

Im Rahmen des vertragszahnärztlichen Versorgungssystems in Deutschland ist eine Inanspruchnahme rein präventiver Maßnahmen im engeren Sinne bisher nur im Kindes- und Jugendalter und durch Pflegebedürftige möglich. Eine Ausnahme bildet lediglich die Zahnsteinentfernung. Für Kinder und Jugendliche stehen die Früherkennungsuntersuchungen bzw. die Individualprophylaxeleistungen zur Verfügung. Für Pflegebedürftige wurden unter anderem 2013 bzw. 2014 spezifische Gebührennummern zur Inanspruchnahme präventiver Maßnahmen geschaffen.

Früherkennungsuntersuchungen für Kinder bis zu 72 Monate wurden bisher noch unzureichend genutzt. Zwischen 2010 (31,9 %) und 2018 (35,2 %) war jedoch ein kontinuierlicher Anstieg der jährlichen Inanspruchnahmeraten zu verzeichnen (Tab. 1; [4]). Allerdings scheint in diesem Bereich noch ein erhebliches ungenutztes Potenzial zur Früherkennung von oralen Erkrankungen und von Risikofaktoren zu bestehen. Individualprophylaktische Maßnahmen zwischen 6 und unter 18 Lebensjahren wurden deutlich häufiger als die Früherkennungsuntersuchungen in Anspruch genommen. Zwischen 2010 (64,0 %) und 2018 (65,4 %) waren die jährlichen Inanspruchnahmeraten eher schwankend; ein klarer Trend war nicht erkennbar (Tab. 1; [4]).

Diagnostische und präventive Maßnahmen bei Pflegebedürftigen wurden von etwa einem Drittel der vollstationär Pflegebedürftigen und mehr als der Hälfte der ambulant Pflegebedürftigen jährlich genutzt. Zwischen 2012 und 2016 blieb die jährliche Inanspruchnahmerate in beiden Fällen etwa gleich. Sie betrug bei vollstationär Pflegebedürftigen um 39 % und bei ambulant Pflegebedürftigen um 55 % (Tab. 1; [6]). Ob der gebührenrechtliche Anreiz zur Erbringung präventiver Leistungen langfristig zu einer Verbesserung der Inanspruchnahme in dieser vulnerablen Gruppe führt, bleibt abzuwarten.

## Ausgaben

Analog zu den Inanspruchnahmen waren auch die Ausgaben hoch. So entfielen im Jahr 2019 in Deutschland 6,3 % der Ausgaben der gesetzlichen Krankenversicherung (GKV) auf die vertragszahnärztliche Versorgung [16]. Im Jahr 2019 entsprach dies nach Aussagen des GKV-Spitzenverbandes einer Summe von 11,5 Mrd. € für zahnärztliche Behandlung und 3,5 Mrd. € für Zahnersatz. Pro versicherte Person wurden im Jahr 2018 im Durchschnitt 193,63 € über alle Bereiche der vertragszahnärztlichen Versorgung hinweg verausgabt [4]. Im Vergleich zwischen alten und neuen Bundesländern waren keine wesentlichen Unterschiede mehr feststellbar (194,53 € neue Bundesländer vs. 193,48 € alte Bundesländer), obwohl die Inanspruchnahmerate in den neuen Bundesländern deutlich höher lag (75,3 % vs. 69,9 %; [4]).

Während die Inanspruchnahmeraten in der vertragszahnärztlichen Versorgung mehr oder weniger stagnierten bzw. leicht zurückgingen, stiegen die Ausgaben der GKV moderat. Bei der Einordnung dieser Zahlen bestehen allerdings erhebliche Unsicherheiten bezüglich der von den Versicherten und ggf. privaten Zusatzversicherungen zu tragenden Kosten und damit auch der Gesamtausgaben. Vielfach sind private Mehrkosten (Mehrkostenvereinbarung Füllungen), Zusatzkosten, die selbst getragen werden müssen (Beispiel Kieferorthopädie) und außervertragliche Leistungen (Beispiel Implantate) üblich. Diese Ausgaben können über die Routinedaten der Kassen nicht oder nur unvollständig abgebildet werden. Die häufig in Anspruch genommene professionelle Zahnreinigung ist in der Regel privat zu bezahlen. Bei prothetischen Leistungen besteht auf der Grundlage des Festzuschusssystems eine erhebliche Zuzahlung, besonders bei Versorgungen jenseits der Regelversorgung (gleichartige und andersartige Versorgung). Im Jahr 2018 schwankten die Eigenanteile bei Zahnersatz zwischen 47,3 % in Sachsen-Anhalt und 66,7 % in Bayern. Implantatchirurgische Kosten waren nicht berücksichtigt. Die Entwicklung der Gesamtausgaben (Kassen- und Eigenanteile) zeigte moderate jährliche Steigerungen von 1,88 % (Regelversorgung), 2,75 % (gleichartige Versorgung) und 2,83 % (andersartige Versorgung). Der Auswertungszeitraum lag zwischen 2012 und 2017 (andersartige Versorgung 2014 bis 2017; [3]).

Die Versorgung des zahnlosen Unterkiefers, der ohne Implantate in vielen Fällen nicht zufriedenstellend versorgt werden kann, ist ein gutes Beispiel für den Einfluss der bei Inanspruchnahme selbst zu tragenden Kosten auf soziale Ungleichheiten und die Teilhabe am medizinischen Fortschritt. Es wurde die Inanspruchnahme von einfachen Vollprothesen und implantatgestützten Prothesen bei Senioren im Jahr 2014 in den Bundesländern untersucht [5]. Der Anteil von Implantatversorgungen betrug 16 % in Bayern und 6 % in Mecklenburg-Vorpommern. Implantatversorgungen werden mit einem niedrigen Festzuschuss bezuschusst, der jedoch angesichts der hohen Gesamtkosten kaum ins Gewicht fällt. Die immer auch auftretenden implantatchirurgischen Kosten sind komplett privat zu tragen. Teilweise können die durch die Krankenkasse nicht getragenen Kosten über private Zusatzversicherungen abgedeckt werden. Daten dazu fehlen. Trotzdem dürften finanzielle Barrieren eine nicht unerhebliche Rolle bei den regionalen Unterschieden bei Implantatversorgungen im zahnlosen Unterkiefer spielen.

## Stärken und Limitationen von Routinedatenanalysen

Die dargestellten Daten zur Inanspruchnahme und zu den Ausgaben basieren auf Abrechnungsdaten einer großen Krankenversicherung. Sie sind gut geeignet, das Versorgungsgeschehen zu beschreiben. Im Gegensatz zu den publizierten Daten der kassenzahnärztlichen Vereinigungen (vgl. [16]) können bei Nutzung der Versicherungsdaten konkrete soziodemografische Zusammenhänge zwischen Inanspruchnahme und Inanspruchnehmenden hergestellt werden. Auch wenn diese Daten keine Bevölkerungsrepräsentativität in engerem Sinne aufweisen, können sie doch näherungsweise als repräsentativ für den Anteil der gesetzlich Versicherten in Deutschland gelten. Der Anteil der etwa 11 % nicht gesetzlich versicherten Menschen in Deutschland ist aus methodischen Gründen nicht Bestandteil dieser Analysen, erscheint vom Gesamtvolumen her jedoch weniger bedeutsam.

Die Interpretation von Routinedatenanalysen sollte stets unter Berücksichtigung ihrer inhärenten Limitationen erfolgen. Aus Routinedaten können die Ausrichtung der vor Ort praktizierten Zahnmedizin und ihre Auswirkungen auf die Mundgesundheit nur in Ansätzen eingeschätzt werden. Gleiches gilt in Abhängigkeit vom Studientyp auch für viele epidemiologische Studien. In Querschnittsstudien gefundene epidemiologische Veränderungen sollten mit der gebotenen Vorsicht interpretiert werden, da sie aufgrund nicht möglicher Kausalitätsnachweise zur Erkennung von Ursache-Wirkungs-Beziehungen, hier beispielsweise bezüglich des Zusammenhanges zwischen zahnmedizinischen Strategien und resultierender Mundgesundheit, ungeeignet sind. Nur mit prospektiven Ansätzen können derartige Informationen auf wissenschaftlicher Basis erlangt werden. Explorativ könnte aus einer Zusammenführung von Abrechnungsdaten mit epidemiologischen Querschnittsdaten eine Verbesserung des Kenntnisstandes erreicht werden, der auch als Grundlage bei der Planung von prospektiven Studien genutzt werden könnte. Den Autoren ist wohl bewusst, dass für die Entscheider im Gesundheitswesen langfristig angelegte Studien und entsprechend erst weit in der Zukunft zu erwartende Ergebnisse wenig passfähig sind. Eine Alternative können empirisch abgeleitete, zielgerichtete Maßnahmen mit entsprechender Begleitforschung sein.

## Fazit und Ausblick auf eine präventionsorientierte Zahnmedizin

Die zahnärztliche Versorgung findet in Deutschland auf einem hohen Inanspruchnahmeniveau statt. Die Ausgaben im System der vertragszahnärztlichen Versorgung stiegen nur moderat. Durch vielfältige privat zu tragende Mehrkosten, private Zusatzleistungen und reine Privatleistungen sind allerdings die tatsächlichen Ausgaben der Versicherten anhand von Routinedaten nicht zu erfassen.

Durch die etablierte regelmäßige Inanspruchnahme bietet die deutsche Versorgungsrealität prinzipiell gute Voraussetzungen für eine präventionsorientierte Zahnmedizin. Auf der anderen Seite birgt sie aber auch ein Potenzial von Überdiagnostik und Übertherapie. Zu derartigen unerwünschten Effekten können aus den Daten allerdings keine Informationen gewonnen werden. Wenn auch eine positive Tendenz zu einer leicht steigenden Inanspruchnahme präventiver Leistungen bei Kindern über die letzten Jahre erkennbar ist, bildet sich nach wie vor auch im deutschen Versorgungsgeschehen eine immer noch mehrheitlich auf invasive Intervention orientierte Zahnmedizin ab. Eine echte Trendumkehr mit einer deutlichen Verlagerung der im Versorgungssystem erbrachten Leistungen in Richtung Prävention und weg von der invasiven Intervention lässt sich aus den vorliegenden Daten nicht ableiten. Wegen des bestehenden Vergütungssystems, das nach wie vor den Schwerpunkt auf Therapieleistungen legt, sollten entsprechende Erwartungen auch nicht zu hoch angesiedelt werden.

Die Gesundheitskompetenz der Bevölkerung gilt es, für die Umsetzung eines Richtungswechsels noch weiterzuentwickeln. Zwar ist ein bereits bestehendes gutes Niveau des Gesundheitsbewusstseins feststellbar [15], allerdings sind im Umgang mit Gesundheitsinformationen auch in der Zahnmedizin noch erhebliche Defizite zu vermuten. Eine repräsentative Studie ergab bei 58,8 % der deutschen Bevölkerung eine geringe Gesundheitskompetenz mit einer Verschlechterung in den letzten 7 Jahren [23].

Bezüglich des Stellenwertes der Prävention in der Versorgungsrealität ist zu berücksichtigen, dass die Präventionsleistungen auch beim gesetzlich Versicherten zu einem erheblichen Anteil nicht in den Abrechnungsdaten erfasst sind, da sie häufig als Privatleistungen erbracht werden. Hier ist vor allem die professionelle Zahnreinigung zu nennen. Insofern liegt bei der Auswertung von Routinedaten eine Unterschätzung präventiver Maßnahmen vor. Die Inanspruchnahme invasiver Therapieleistungen bleibt auf der Basis der abgerechneten Morbidität jedoch hoch. Als sinnvolle Instrumente, um einen wirklich substanziellen Paradigmenwechsel umzusetzen, können entsprechende Modifikationen und Anreize im Versorgungssystem in Verbindung mit einer konsequenten Umsetzung von qualitätsrelevanten Maßnahmen angesehen werden. In diesem Kontext denkbar wären die Verbesserung des Zuganges zur Individualprophylaxe für Erwachsene innerhalb der GKV, eine Veränderung des wertmäßigen Verhältnisses im BEMA zugunsten präventiver und wenig invasiver Maßnahmen und die Begünstigung sekundärpräventiv ausgerichteter Konzepte im Zahnersatzbereich, zum Beispiel Vermeidung von medizinisch nicht notwendigem Zahnersatz [24].
